# A novel Iowa–Mayo validated composite risk assessment tool for allogeneic stem cell transplantation survival outcome prediction

**DOI:** 10.1038/s41408-021-00573-6

**Published:** 2021-11-20

**Authors:** Kalyan Nadiminti, Kimberly Langer, Ehsan Shabbir, Mehrdad Hefazi, Lindsay Dozeman, Yogesh Jethava, Bradley Loeffler, Hassan B. AlKhateeb, Mark Litzow, Mrinal Patnaik, Mithun Shah, William Hogan, Umar Farooq, Margarida Silverman, Sarah L. Mott

**Affiliations:** 1 Division of Hematology, University of Wisconsin, Madison, WI 53792 USA; 2 Division of Hematology, Mayo Clinic, Rochester, MN 55905 USA; 3 Department of Medicine and Neurology, Wright State University, Dayton, OH 45435 USA; 4 Division of Hematology, Blood and Marrow Transplantation, University of Iowa Hospitals and Clinics, Iowa City, IA 52242 USA; 5 Holden Comprehensive Cancer Center, University of Iowa, Iowa City, IA 52242 USA

**Keywords:** Chemotherapy, Stem-cell therapies

## Abstract

Allogeneic hematopoietic stem cell transplantation (HSCT) is a curative option for many hematologic conditions and is associated with considerable morbidity and mortality. Therefore, prognostic tools are essential to navigate the complex patient, disease, donor, and transplant characteristics that differentially influence outcomes. We developed a novel, comprehensive composite prognostic tool. Using a lasso-penalized Cox regression model (n = 273), performance status, HCT-CI, refined disease-risk index (rDRI), donor and recipient CMV status, and donor age were identified as predictors of disease-free survival (DFS). The results for overall survival (OS) were similar except for recipient CMV status not being included in the model. Models were validated in an external dataset (n = 378) and resulted in a c-statistic of 0.61 and 0.62 for DFS and OS, respectively. Importantly, this tool incorporates donor age as a variable, which has an important role in HSCT outcomes. This needs to be further studied in prospective models. An easy-to-use and a web-based nomogram can be accessed here: https://allohsctsurvivalcalc.iowa.uiowa.edu/.

## Introduction

Allogeneic hematopoietic stem cell transplantation (HSCT) remains an indispensable curative therapy for several malignant and nonmalignant hematologic conditions. The outcomes of HSCT have improved over the years due to advances in supportive care and therapeutic modalities. Additionally, our ability to predict patients who are at a higher risk of adverse outcomes related to disease and/or transplant characteristics, and thereby individualize treatments, continues to be refined. Traditional outcome predictors in HSCT are patient age, comorbidity risk, disease status, HLA- and ABO-matching disparities, and other host- and disease-related factors [1–5].

Several tools have been published to inform critical decisions in HSCT, including risk of relapse post-HSCT, nonrelapse mortality (NRM), and overall survival (OS). These tools are also helpful to stratify patients according to relative risks imparted by these independent disease-related and patient characteristics [6, 7]. Additionally, they guide us when counseling patients and help physicians individualize transplant management for the patients.

The most widely used prognostic tool is the Hematopoietic Cell Transplantation specific Comorbidity Index (HCT-CI) and HCT-CI/age, which are adapted from Charlton Comorbidity Index (CCI) for assessment of HSCT patients, and has been validated in a large dataset [6, 7]. These indexes are primarily used to objectively assess organ function status and predict NRM and OS. Disease Risk Index (DRI) or refined DRI (rDRI) predicts OS primarily based on the type and status of disease prior to HSCT [8, 9]. A number of other multivariable tools in use are the European Group for Bone Marrow Transplantation (EBMT) risk score [10], pretransplant assessment of mortality (PAM) [11], and more recently, the acute leukemia—EBMT (AL-EBMT) model [12] and a composite hematopoietic cell-transplant composite-risk (HCT-CR) model [13]. These prediction tools differ from one another with respect to composition of variables, disease groups studied, end points, model building, validation, and calibration methodologies [14]. The c-statistic, by which most of the prognostic tools are built to discriminate patients, also varies between tools, at least partly dependent on the variables incorporated. Additionally, many advances have occurred with respect to identification of important variables, therapeutic modalities, and treatment selection over the years, which were not accounted for in most of the existing older models. Therefore, there is a constant effort to improve and develop holistic prognostic scoring systems as newer variables of significance, and statistical methods are identified.

In this study, we hypothesized that integration of more contemporarily used recipient, donor, and transplant characteristics would improve prediction of post-transplant survival outcomes compared with the currently published tools. After model building, we validated our tool in an external patient dataset and the results are presented here.

### Patients and methods

This study includes two cohorts of patients from the University of Iowa Health Care (UIHC) and Mayo Clinic (MC). Patients ≥18 years of age who received first HSCT from a peripheral blood stem cell (PBSC) source for any malignant hematologic indication between 2010 and 2016 from HLA-matched related (MRD), HLA-matched unrelated (MUD), HLA-mismatched unrelated (MMUD), and HLA-mismatched related donors (MMRD/haploidentical) donors were included. HLA matching at -A, _B, -C, and -DRB1 loci was defined as matched status.

Patients with HSCT from bone marrow source and those with incomplete or missing data were excluded. After obtaining IRB approval from the respective institutions, we collected demographic, clinical, and outcome data.

### Endpoints and definitions

The primary endpoints used for the models were two-year disease-free survival (DFS) defined as time from the initial allogeneic transplant to relapse or death due to HCT-related causes, and two-year overall survival (OS) defined as time from the initial allogeneic transplant to death due to any cause. Patients alive and without relapse at two years were censored.

The intensity of conditioning regimens was defined as per Bacigalupo et al [15]. HCT-CI and rDRI were defined as previously described per Sorror et al. and Armand et al. [6, 9].

### Statistical analysis

The training dataset included 273 patients treated at UIHC, and the external testing dataset included 348 patients treated at MC.

Using the training dataset, a lasso-penalized Cox regression model was applied to identify prognostic predictors of two-year DFS and OS. Predictors under consideration included: recipient (age <55 vs 55 + , sex, KPS < 90 vs 90 + , HCT-CI, ABO type, and CMV status), disease (type, rDRI), donor (age <30 vs 30 + , sex, ABO type, and CMV status), and transplant (preparative regimen, year of transplant, related/unrelated, and match/mismatch) characteristics. The lasso penalty parameter was derived as the mean of 1000 iterations of 10-fold cross-validation. Median and IQR time-dependent area under the curve (AUC) using 1000 bootstrap samples was obtained using the method proposed by Uno et al. [16] to assess internal model validation. To assess internal model calibration, a risk score was computed from the regression coefficients. Patients were stratified based on a median cut point of risk scores.

We used Harrell’s concordance index (c-index) in which a c-index of 1.0 indicates a model’s discriminatory function to be perfect, while a score of 0.50 indicates a discrimination function not dissimilar to chance alone.

Differences in two-year DFS and OS between risk strata were evaluated using a log-rank test. Optimism-corrected (1000 bootstrap samples) predicted survival probabilities were compared with observed survival probabilities at two years. Median-predicted survival probabilities were plotted against the median observed survival probabilities along with 95% confidence interval estimated by the Kaplan–Meier method for each risk strata.

The model derived in the building phase was applied to the testing dataset. External model validation was assessed by constructing a time-dependent ROC curve. To assess external model calibration, patients were stratified by risk score. Two-year DFS and OS differences between risk strata were evaluated using a log-rank test. Additionally, median predicted survival probabilities were plotted against the median observed survival probabilities at two years along with the 95% confidence interval estimated by the Kaplan–Meier method for each risk strata.

All analyses were conducted using SAS v9.4 (SAS Institute, Cary, NC) or R (www.r-project.org) and the glmnet package [17] and the hdnom package [18].

## Results

### Comparison of patient cohorts

The baseline and transplant clinical characteristics are in Table 1. The UIHC cohort included 273 patients who received their first HSCT between 2010 and 2015 and the MC cohort included 348 patients who received their first HSCT between 2010 and 2016.

**Table 1:** Patient demographic and transplant characteristics of the two cohorts.

			Group		
Covariate	Statistics	Level	UIHC N = 273	Mayo N = 348	*P*-value
Time Since Diagnosis	N (Col %)	6+ months	136 (49.8)	167 (48.0)	0.65
	N (Col %)	<6 months	137 (50.2)	181 (52.0)	
Age	N (Col %)	55+	144 (52.7)	193 (55.5)	0.50
	N (Col %)	<55	129 (47.3)	155 (44.5)	
Sex	N (Col %)	F	111 (40.7)	134 (38.5)	0.59
	N (Col %)	M	162 (59.3)	214 (61.5)	
Disease	N (Col %)	Acute lymphoblastic leukemia (ALL)	46 (16.8)	48 (13.8)	**<0.01**
	N (Col %)	Acute myelogenous leukemia (AML)	119 (43.6)	172 (49.4)	
	N (Col %)	Myelodysplastic/myeloproliferative diseases (MDS/MPN)	60 (22.0)	80 (23.0)	
	N (Col %)	Non-Hodgkin lymphoma (NHL)	18 (6.6)	4 (1.1)	
	N (Col %)	Other	30 (11.0)	44 (12.6)	
Performance Status at Transplant	N (Col %)	KPS 90+	178 (65.2)	214 (61.5)	0.34
	N (Col %)	KPS < 90	95 (34.8)	134 (38.5)	
Disease Risk Index	N (Col %)	High–Very High	97 (35.5)	71 (20.4)	**<0.01**
	N (Col %)	Intermediate	116 (42.5)	189 (54.3)	
	N (Col %)	Low	60 (22.0)	88 (25.3)	
Comorbidity Index	N (Col %)	High	192 (70.3)	94 (27.0)	**<0.01**
	N (Col %)	Intermediate	56 (20.5)	165 (47.4)	
	N (Col %)	Low	25 (9.2)	89 (25.6)	
Regimen	N (Col %)	Myeloablative	199 (72.9)	223 (64.1)	**0.02**
	N (Col %)	RIC/Nonmyeloablative	74 (27.1)	125 (35.9)	
Year of Transplant	N (Col %)	2010	40 (14.7)	36 (10.3)	**<0.01**
	N (Col %)	2011	41 (15.0)	61 (17.5)	
	N (Col %)	2012	43 (15.8)	63 (18.1)	
	N (Col %)	2013	32 (11.7)	64 (18.4)	
	N (Col %)	2014	41 (15.0)	65 (18.7)	
	N (Col %)	2015	32 (11.7)	59 (17.0)	
	N (Col %)	2016	0 (0)	44 (16.1)	
ABO	N (Col %)	A	113 (41.4)	140 (40.2)	0.99
	N (Col %)	AB	11 (4.0)	15 (4.3)	
	N (Col %)	B	31 (11.4)	39 (11.2)	
	N (Col %)	O	118 (43.2)	154 (44.3)	
CMV	N (Col %)	N	60 (22.0)	103 (29.6)	**0.03**
	N (Col %)	P	213 (78.0)	245 (70.4)	
Transplant Type	N (Col %)	Related	107 (39.2)	200 (57.5)	**<0.01**
	N (Col %)	Unrelated	166 (60.8)	148 (42.5)	
Match/Mismatch	N (Col %)	Match	229 (83.9)	335 (96.3)	**<0.01**
	N (Col %)	Mismatch	44 (16.1)	13 (3.7)	
Donor Age	N (Col %)	30+	140 (51.3)	250 (71.8)	**<0.01**
	N (Col %)	<30	133 (48.7)	98 (28.2)	
Donor Sex	N (Col %)	F	95 (34.8)	115 (33.0)	0.65
	N (Col %)	M	178 (65.2)	233 (67.0)	
Donor ABO/Rh	N (Col %)	A	111 (40.7)	136 (39.1)	0.19
	N (Col %)	AB	14 (5.1)	15 (4.3)	
	N (Col %)	B	20 (7.3)	44 (12.6)	
	N (Col %)	O	128 (46.9)	153 (44.0)	
Donor CMV	N (Col %)	N	178 (65.2)	187 (53.7)	**<0.01**
	N (Col %)	P	95 (34.8)	161 (46.3)	

Where the *p* values indicate the differences in the characteristics between the two cohorts, and significant differences are noted as bold.

Disease, DRI, HCT-CI, regimen, year of transplant, recipient CMV status, transplant type and match, and donor age and CMV status significantly differed between cohorts. Notably, acute myelogenous leukemia (AML) was the most common indication present in 43.6% and 49.4%, followed by myelodysplastic syndrome (MDS) and myeloproliferative neoplasm (MPN) in 22.0% vs 23.0% and acute lymphoblastic leukemia (ALL) in 16.8% vs 13.8% in UIHC and MC cohorts, respectively. Other diagnosis category consisted of UIHC cohort: 18 chronic myeloid leukemia (CML); 1 Hodgkin lymphoma (HL); 10 other leukemia (OL); and 1 plasma cell disease (PCD); MC cohort: 2 chronic lymphocytic leukemia (CLL); 11 CML; 31 OL.

There were more patients in UIHC cohort with high–very high DRI (35.5% vs 20.4%), high HCT-CI scores (70.3% vs 27.0%), and those who received a myeloablative regimen (72.9% vs 64.1%) in UIHC compared with MC, respectively. On the other hand, more patients in MC received a related donor (57.5% vs 39.2%), matched (MSD or MUD) donor (96.3% vs 83.9%), and donors with age greater than 30 years (71.8% vs 51.3%) compared with UIHC.

### Outcomes

**Fig. 1:**
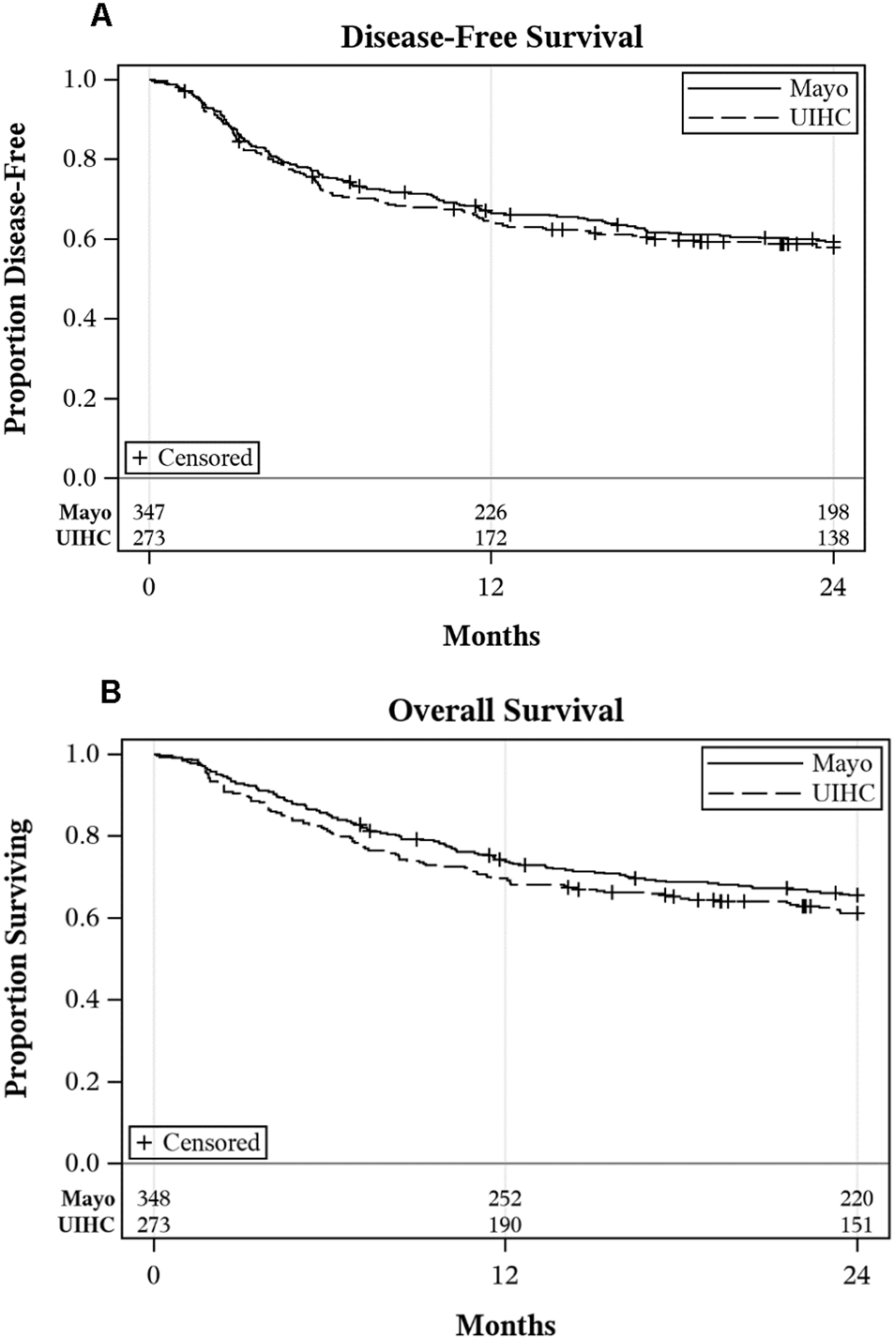
Two-year disease-free survival and overall survival for the training (UIHC) and testing (Mayo Clinic) cohorts. Figure 1 represents two-year DFS **A** and two-year OS **B** for the UIHC and Mayo Clinic cohorts which were 58% and 59%, and 61% and 66%, respectively. Disease-free survival (DFS) is defined as time from the initial allogeneic transplant to relapse or death due to HCT-related causes; overall survival (OS) defined as time from the initial allogeneic transplant to death due to any cause. Patients alive and without relapse at two years were censored.

Two-year DFS was 58% and 59% and 2-year OS was 61% and 66% for UIHC and Mayo cohorts, respectively (Fig. 1).

### Two-year disease-free survival

**Fig. 2:**
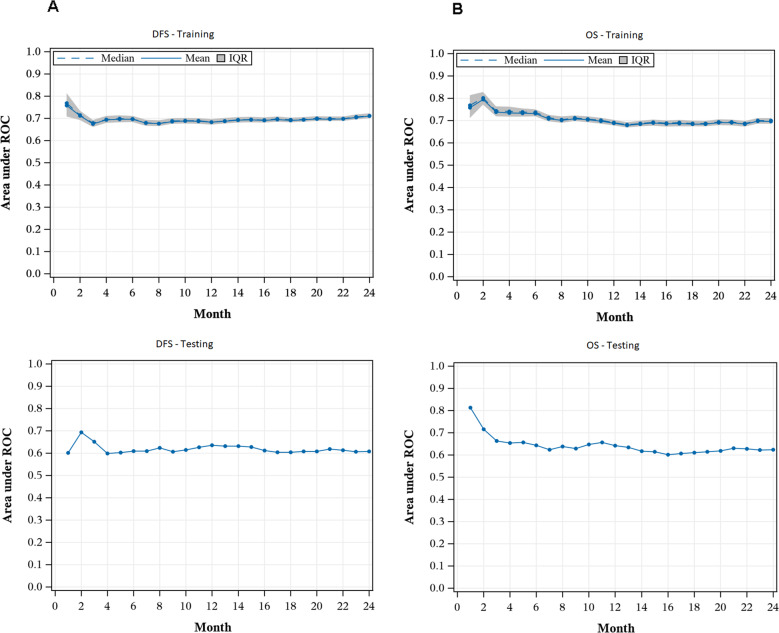
Time-dependent AUC for disease-free and overall survival. **A** Panels show time-dependent AUC values for disease-free survival in the training and testing cohorts, at 0.71 and 0.61, respectively. **B** Panels show time-dependent AUC values for overall survival in the training and testing cohorts, at 0.70 and 0.61, respectively.

**Fig. 3:**
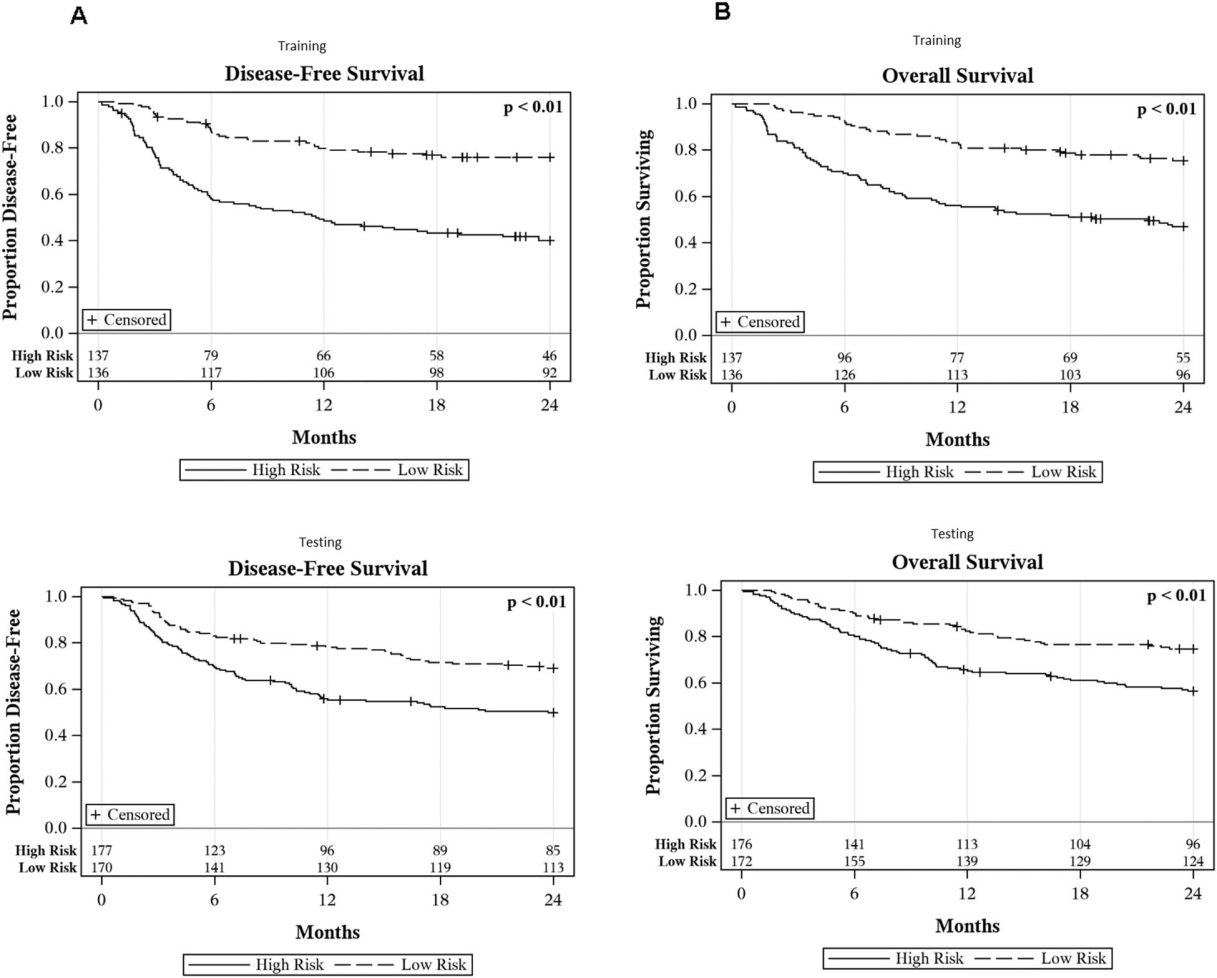
Risk-stratified 2-year disease-free survival and overall survival. **A** Panels show significant difference in two-year DFS between low and high-risk patients stratified by the model at 76% and 40% in the training and 69% and 50% in the testing sets, respectively. **B** Panels show significant difference in two-year OS between the low- and high-risk patients stratified by the model at 76% and 47% and 75% and 56% among training and testing sets, respectively.

After application of the lasso-penalized Cox regression model, the final model included the following variables: performance status, disease-risk index, comorbidity index, patient CMV status, donor CMV status, and donor age (Table 2). Median AUC for the prediction of two-year DFS in the training set was 0.71 (IQR 0.70–0.72), demonstrating good internal discrimination (Fig. 2A). Additionally, AUC across time was relatively consistent between 1- and 2 years post transplant. Internal model calibration showed good agreement between observed and predicted survival probabilities (Supplementary Fig. 1A), which is further supported by a significant difference in DFS between risk groups (*p* < 0.01). Two-year DFS was 76% and 40% for low and high risk, respectively (Fig. 3A).

**Table 2:** Variables selected for the model.

		Disease-free survival	Overall survival
Covariate	Level	Hazard ratio	Hazard ratio
Performance Status	90+	0.77	0.67
	<90	Ref	Ref
Disease Risk Index	Low	0.60	0.62
	Intermediate/High	Ref	Ref
Comorbidity Index	Low	0.77	0.94
	Intermediate/High	Ref	Ref
Donor Age	30+	1.23	1.03
	<30	Ref	Ref
CMV	Positive	1.01	–
	Negative	Ref	–
Donor CMV	Positive	0.97	0.89
	Negative	Ref	Ref

After applying the final model to the testing dataset, AUC was 0.61 at two years with AUC remaining consistent between 1- and 2 years post transplant (Fig. 2A). External model calibration showed good agreement (Supplementary Fig. 1A), which is further supported by a significant difference in DFS by risk groups (*p* < 0.01). Two-year DFS of 69% and 50% for low and high-risk groups, respectively (Fig. 3A).

### Two-year overall survival

After application of the lasso-penalized Cox regression model, the final model included the following variables: performance status, disease-risk index, comorbidity index, donor CMV status, and donor age (Table 2). Median AUC for the prediction of two-year OS was 0.70 (IQR 0.69-0.71) in the training set demonstrating good internal discrimination (Fig. 2B). Additionally, AUC across time was relatively consistent between 1- and 2-years post transplant. Internal model calibration showed good agreement between observed and predicted survival probabilities (Supplementary Fig. 1B) which is further supported by a significant difference in OS between risk groups (*p* < 0.01). Two-year OS was 76% and 47% for low and high risk, respectively (Fig. 3B).

After applying the final model to the testing dataset, AUC was 0.61 at two years with AUC remaining consistent between 1- and 2 years post transplant (Fig. 2B). External model calibration showed good agreement (Supplementary Fig. 1B), which is further supported by a significant difference in OS by risk groups (*p* < 0.01). Two-year OS was 75% and 56% for low and high risk, respectively (Fig. 3B).

## Discussion

In this study, we present a new, externally validated composite prognostic tool for hematologic malignancies to predict two-year DFS and OS following HSCT. For two-year DFS, performance status, HCT-CI and rDRI of the patient, CMV status of patient and donor, and age of the donor had significant impact. Model discrimination assessed by the c-statistic was 0.71 and 0.62 in the training and testing datasets, respectively, for two-year DFS. The results for two-year OS were similar, except that patient CMV status was not included in the final model. Additionally, model calibration showed good agreement between the predicted and observed outcomes in the training and test cohorts demonstrating a consistent performance of the tool in the prediction of outcomes. Finally, using this model, we could discriminate patient cohorts into 2 distinct risk groups with significantly different two-year DFS and OS rates. The high-risk group had a significantly lower two-year OS of 56% compared with 75% in the lower-risk group. An important feature of our model is that it captures the most crucial pretransplant recipient, disease, and donor characteristics that are known to influence transplant outcomes with a c-statistic of 0.62.

Of the numerous variables that are conventionally used to assess risk, age, performance status, and comorbidity burden of the patient remain the foremost and powerful prognostic factors in oncology, including in HSCT [19].

Various models have studied several important variable factors in combination for prediction of outcomes. This important differences between various existing tools compared with our model, including the endpoints used for predictions are summarized in Table 3.

**Table 3:** Comparison of allogeneic HSCT prognostic scoring systems.

	HCT-CI [6]	rDRI [9]	EBMT [10]	PAM [11]	AL-EBMT [12]	HCT-CR [31]	Iowa/Mayo
Years of transplantation	1997–2003	2008–2010	1990–2005	1990–2002	2000–2011	2010–2016	2000–2016
Disease included	AML, ALL, CML, CLL, MDS, MM, NHL, HD, AA, Nonmalignant indications	MPN, HD, NHL, T-cell NHL, CLL, MCL, CML, AML, BL, ALL, MDS, MM	AML, ALL, CML, MDS, MM, NHL, AA	AML, ALL, CML, CLL, MDS, MM, NHL, HL, AA, nonmalignant	AML, ALL	AML, ALL, MDS, NHL, CLL, CML, HL, MPN and MM	ALL, AML, MDS MPN, NHL, Other
Primary endpoint	NRM	OS	OS	OS	100-day mortality	OS	OS
Secondary endpoints	OS	None	NRM and relapse	None		DFS, GRFS, NRM and RI	DFS
AUC/C-statistic for OS in testing cohort	0.66	0.63	0.62	0.69	0.70	0.69	0.62
Total sample size	1055	13,131	56,505	2802	28,236	1447	651
Sample size training/testing	708/347	9,849/2,382	NA	1,401/1,401	19,765/8,47 1	NA	273/378
Major variables included in the final tool	13 system comorbidities	Disease, disease status at HSCT, karyotype (AML and MDS)	Disease stage, donor type (MRD or MUD), age of recipient, time from HSCT, sex match of recipient and donor	Age of recipient (<50, 50–60, >60), donor type (MRD or MUD), disease risk conditioning regimen, renal disease, hepatic disease, pulmonary disease	Disease stage, KPS, Age of recipient (<37, ≥37), Time from diagnosis to HSCT, Donor type (MSD or MUD), Year of HSCT (2003–2003, subsequent) Annual allo- HSCT center experience, donor and recipient CMV serostatus	HCT-CI/age, rDRI	KPS, HCT-CI, rDRI, donor age, CMV serostatus
Model development methods used in original publication	Cox regression	Cox regression	Cox regression	Cox regression	Nonparametric data mining approach	Cox regression	Cox regression
Training and validation cohort derivation in the original model	Randomly split internal cohort	Randomly split internal cohort	Randomly split internal cohort	Randomly split internal cohort	Randomly split internal cohort	Independent internal cohort	Independent external cohort
Donor type included	MRD MUD	MRD MUD	MRD MUD	MRD MUD	MRD MUD	MRD MUD MMRD MMUD	MRD MUD MMRD MMUD

CMV serostatus of donor and recipient remains a significant determinant of important HSCT outcomes such as DFS and OS, beyond the direct impact on CMV reactivation-associated morbidity and mortality [20]. Additionally, few studies suggested a likely favorable role of positive CMV serostatus of donor and/or recipient on early immune reconstitution [21], and reduced relapses [22].

ABO matching between the recipient and donors is another critical variable that is considered during donor selection. There have been conflicting reports about the impact of the ABO mismatching and outcomes of HCT. While some major registry and single institutional studies showed an adverse impact on increased GVHD, NRM, or OS [23–26], few other studies, including this recent analysis, did not find any major impact on the outcomes [27]. In our model, ABO status of the recipient and donor was not found as a significant variable for DFS or OS prediction.

These variables were studied, either independently or in combination, by multiple predictive tools.

HCT-CI and Comorbidity-age Index (HCT-CI that accounts for the age of the patient) were the first of the prognostic tools developed to estimate NRM and OS [6, 28]. Despite many attempts to augment the predictability [29], no major improvements in the c-statistic were noted [28] and the original HCT-CI still remains one of the most widely used tools in HSCT prognostication of NRM. Alternatively, rDRI was developed to estimate OS based primarily on the risk of relapse of the disease, regardless of the conditioning intensity, recipient age, and donor type and discriminates 4 distinct groups [9]. The discrimination function of HCT-CI and rDRI for OS is reported to be 0.63 and 0.66, respectively, in the original publications [6, 9]. Composite models have been developed in an attempt to improve discrimination and predictability by integrating various recipient, donor, and transplant characteristics. Of those, EBMT and PAM are prominent validated tools that have evaluated OS as a primary endpoint in various groups of hematologic malignancies and the c-statistics for OS are 0.62 and 0.69, respectively [10, 11]. However, they both miss important characteristics such as performance status and CMV serostatus. Although disease stage was included in both, the criteria of staging were not uniform nor validated as rDRI was not available at the time of development of these tools.

A more recently developed HCT-CR showed a relatively better c-statistic of 0.69 [30]. HCT-CR is a composite model that combined rDRI and HCT-CI/age with the reported superior ability to estimate NRM and OS, and to stratify 4 risk groups with significantly different three-year median OS [13, 31]. While their original model was only restricted to AML and MDS patients, the validation study was performed on an independent internal dataset and expanded to multiple disease groups and other outcomes such as GVHD and relapse-free survival (GRFS) [31], but this tool needs to be externally validated.

Most tools predict OS and NRM, while our current model and the HCT-CR also evaluate DFS. On the other hand, AL-EBMT is a model derived from machine-learning (ML) algorithm, restricted to AML and ALL patients only and the primary endpoint was 100-day mortality with a c-statistic of 0.70 [32].

Another distinction of our tool is inclusion of donor age. In recent years, age of the donor has been reported as one of the most influential factors on post-transplant outcomes. In large registry studies, younger donor age correlated with improved outcomes, including overall survival, which was noted across unrelated and haploidentical donor groups [33–35]. Although similar trends were reported by a few other studies [36, 37], an institutional study did not necessarily show a differential impact of donor age when dichotomized at 60 years [38]. Our tool is the first validated multivariable model that incorporates donor age, and provides further evidence for younger age of the donor as an emerging predictive variable for DFS and OS after HSCT for various hematologic malignancies.

External validation of scoring systems is important to assess the generalizability of any prognostic tool. In this regard, an important strength of our study is that, in compliance with TRIPOD guidelines [39, 40], model calibration showed agreement between observed and predicted outcomes and validation performed in an independent, external dataset, and showed a minimal decline in discrimination relative to internally validated values.

There could be specific limitations to generalizability even for validated models that are particularly highlighted in external validation studies. For example, in one single-center report, rDRI could not accurately predict OS and PFS in a cohort with a shorter follow-up [41], while another single-center analysis revealed diminished prediction accuracy of HCT-CI when applied to different donor groups [42]. Similarly, inconsistencies were noted for other tools in subsequent external validation studies [43].

Shouval et al in a recent study externally validated and compared performances of various prediction tools in HSCT [44], and appropriately point out that most models in the field of HSCT have at best, modest discrimination function, likely due to various unpredictable complications, and due to our inability to account for all aspects that could influence outcomes [44].

Last, using our model, we were able to discriminate patient cohorts into two distinct risk groups with significantly different 2-year DFS and OS rates. The high-risk group had a significantly lower two-year OS of 56% compared with 75% in the lower-risk group. This information would be helpful for estimation of OS pretransplant and may aid in further preemptive management post HSCT.

In our study, there were some differences between the two datasets. There were more patients with higher risk by rDRI and HCT-CI, and ALL subgroup in the training data (UIHC cohort), while the testing dataset (Mayo cohort) had more patients with significantly older donors. Similarly, differences in center practices relating to transplantation methodologies and donor composition may have also influenced generalizability as demonstrated by the decline in the c-statistic between training and testing datasets. There were differences in the timeframe, cohort sizes, and follow-up duration among the training and testing cohorts that could have also impacted the results [45].

An important strength of our study is that it allows physicians to predict two-year OS and DFS for HSCT with a c-index of 0.62, by combining the most used and validated variables and risk scores representing patient (age, CMV, KPS, and HCT-CI), disease (rDRI), and donor (age and CMV) characteristics. Incorporation of donor age, which is believed to be a formidable contributor to the outcome of transplantation, is an added strength of this tool. Furthermore, TRIPOD guidelines were followed for external validation and calibration attesting to the integrity of the model. The model is easy to use, and a web-based nomogram can be accessed here: https://allohsctsurvivalcalc.iowa.uiowa.edu/.

A few considerable limitations of this study include model building using retrospectively collected data, restriction to PBSC stem cell source, fewer numbers representing some disease groups, such as multiple myeloma, and fewer haploidentical and alternative donor transplants.

The endpoints of interest, target diseases, and the risk factors used in the original model building will have to be considered while applying any prediction tool(s) to a local dataset.

Validation of this tool in other external datasets and continuous refinement with incorporation of validated global prognostic variables, such as fragility index, cognitive assessment of patients, and biomarker correlates, are expected to further improve prognostic value.

## Supplementary information

supplementary figure

## References

[1] Byrd JC (2002). Pretreatment cytogenetic abnormalities are predictive of induction success, cumulative incidence of relapse, and overall survival in adult patients with de novo acute myeloid leukemia: Results from cancer and leukemia group B (CALGB 8461). Blood.

[2] Behrendt CE, Rosenthal J, Bolotin E, Nakamura R, Zaia J, Forman SJ (2009). Donor and Recipient CMV Serostatus and Outcome of Pediatric Allogeneic HSCT for Acute Leukemia in the Era of CMV-Preemptive Therapy. Biol Blood Marrow Transplant.

[3] Craddock C, Szydlo RM, Dazzi F, Olavarria E, Cwynarski K, Yong A (2001). Cytomegalovirus seropositivity adversely influences outcome after T-depleted unrelated donor transplant in patients with chronic myeloid leukaemia: The case for tailored graft-versus-host disease prophylaxis. Br J Haematol.

[4] Craddock C, Labopin M, Pillai S, Finke J, Bunjes D, Greinix H (2011). Factors predicting outcome after unrelated donor stem cell transplantation in primary refractory acute myeloid leukaemia. Leukemia.

[5] Yakoub-Agha I, Mesnil F, Kuentz M, Boiron JM, Ifrah N, Milpied N (2006). Allogeneic marrow stem-cell transplantation from human leukocyte antigen-identical siblings versus human leukocyte antigen-allelic-matched unrelated donors (10/10) in patients with standard-risk hematologic malignancy: a prospective study from the French. J Clin Oncol.

[6] Sorror ML, Maris MB, Storb R, Baron F, Sandmaier BM, Maloney DG (2005). Hematopoietic cell transplantation (HCT)-specific comorbidity index: a new tool for risk assessment before allogeneic HCT. Blood.

[7] Sorror ML, Sandmaier BM, Storer BE, Maris MB, Baron F, Maloney DG (2007). Comorbidity and disease status-based risk stratification of outcomes among patients with acute myeloid leukemia or myelodysplasia receiving allogeneic hematopoietic cell transplantation. J Clin Oncol.

[8] Armand P, Deeg HJ, Kim HT, Lee H, Armistead P, de Lima M (2010). Multicenter validation study of a transplantation-specific cytogenetics grouping scheme for patients with myelodysplastic syndromes. Bone Marrow Transplant.

[9] Armand P, Kim HT, Logan BR, Wang Z, Alyea EP, Kalaycio ME (2014). Validation and refinement of the Disease Risk Index for allogeneic stem cell transplantation. Blood.

[10] Gratwohl A, Stern M, Brand R, Apperley J, Baldomero H, de Witte T (2009). Risk score for outcome after allogeneic hematopoietic stem cell transplantation: a retrospective analysis. Cancer.

[11] Parimon T, Au DH, Martin PJ, Chien JW (2006). A risk score for mortality after allogeneic hematopoietic cell transplantation. Ann Intern Med.

[12] Shouval R, Labopin M, Bondi O, Mishan-Shamay H, Shimoni A, Ciceri F (2015). Prediction of allogeneic hematopoietic stem-cell transplantation mortality 100 days after transplantation using a machine learning algorithm: a European group for blood and marrow transplantation acute leukemia working party retrospective data mining stud. J Clin Oncol.

[13] Kongtim P, Parmar S, Milton DR, Perez J, Rondon G, Chen J (2019). Impact of a novel prognostic model, hematopoietic cell transplant-composite risk (HCT-CR), on allogeneic transplant outcomes in patients with acute myeloid leukemia and myelodysplastic syndrome. Bone Marrow Transplant.

[14] Potdar R, Varadi G, Fein J, Labopin M, Nagler A, Shouval R (2017). Prognostic scoring systems in allogeneic hematopoietic stem cell transplantation: where do we stand?. Biol Blood Marrow Transplant.

[15] Bacigalupo A, Ballen K, Rizzo D, Giralt S, Lazarus H, Ho V (2009). Defining the intensity of conditioning regimens: working definitions. Biol Blood Marrow Transplant.

[16] Uno H, Cai T, Tian L, Wei LJ (2007). Evaluating prediction rules for t-year survivors with censored regression models evaluating prediction rules for f-year survivors with censored regression models. J Am Stat Assoc.

[17] Friedman J, Hastie T, Tibshirani R (2010). Regularization paths for generalized linear models via coordinate descent. J Stat Softw.

[18] Xiao N, Xu Q-S, Li M-Z. hdnom: Building Nomograms for Penalized Cox Models with high-dimensional survival data. 10.1101/065524.

[19] Artz AS, Pollyea DA, Kocherginsky M, Stock W, Rich E, Odenike O (2006). Performance status and comorbidity predict transplant-related mortality after allogeneic hematopoietic cell transplantation. Biol Blood Marrow Transplant.

[20] Schmidt-Hieber M, Labopin M, Beelen D, Volin L, Ehninger G, Finke J (2013). CMV serostatus still has an important prognostic impact in de novo acute leukemia patients after allogeneic stem cell transplantation: a report from the acute leukemia working party of EBMT. Blood.

[21] Ogonek J, Varanasi P, Luther S, Schweier P, Kühnau W, Göhring G (2017). Possible impact of cytomegalovirus-specific CD8+ T cells on immune reconstitution and conversion to complete donor chimerism after allogeneic stem cell transplantation. Biol Blood Marrow Transplant.

[22] Inagaki J, Noguchi M, Kurauchi K, Tanioka S, Fukano R, Okamura J (2016). Effect of cytomegalovirus reactivation on relapse after allogeneic hematopoietic stem cell transplantation in pediatric acute leukemia. Biol Blood Marrow Transplant.

[23] Kimura F, Sato K, Kobayashi S, Ikeda T, Sao H, Okamoto S (2008). Impact of AB0-blood group incompatibility on the outcome of recipients of bone marrow transplants from unrelated donors in the Japan Marrow Donor Program. Haematologica.

[24] Logan AC, Wang Z, Alimoghaddam K, Wong RM, Lai T, Negrin RS (2015). ABO mismatch is associated with increased nonrelapse mortality after allogeneic hematopoietic cell transplantation. Biol Blood Marrow Transplant.

[25] Finke J, Bethge WA, Schmoor C, Ottinger HD, Stelljes M, Zander AR (2009). Standard graft-versus-host disease prophylaxis with or without anti-T-cell globulin in haematopoietic cell transplantation from matched unrelated donors: a randomised, open-label, multicentre phase 3 trial. Lancet Oncol.

[26] Michallet M, Le QH, Mohty M, Prébet T, Nicolini F, Boiron JM (2008). Predictive factors for outcomes after reduced intensity conditioning hematopoietic stem cell transplantation for hematological malignancies: a 10-year retrospective analysis from the Société Française de Greffe de Moelle et de Thérapie Cellulaire. Exp Hematol.

[27] Damodar S, Shanley R, MacMillan M, Ustun C, Weisdorf D (2017). Donor-to-Recipient ABO mismatch does not impact outcomes of allogeneic hematopoietic cell transplantation regardless of graft source. Biol Blood Marrow Transplant.

[28] Sorror ML, Storb RF, Sandmaier BM, Maziarz RT, Pulsipher MA, Maris MB (2014). Comorbidity-age index: a clinical measure of biologic age before allogeneic hematopoietic cell transplantation. J Clin Oncol.

[29] Vaughn JE, Storer BE, Armand P, Raimondi R, Gibson C, Rambaldi A (2015). Design and validation of an augmented hematopoietic cell transplantation-comorbidity index comprising pretransplant ferritin, albumin, and platelet count for prediction of outcomes after allogeneic transplantation. Biol Blood Marrow Transplant.

[30] Dreger P, Sureda A, Ahn KW, Eapen M, Litovich C, Finel H (2019). PTCy-based haploidentical vs matched related or unrelated donor reduced-intensity conditioning transplant for DLBCL. Blood Adv.

[31] Ciurea SO, Kongtim P, Hasan O, Ramos Perez JM, Torres J, et al. Validation of a Hematopoietic Cell Transplant - Composite Risk (HCT-CR) model for post transplant survival prediction in patients with hematologic malignancies. *Clin Cancer Res.* 2020; 26:2404–10. 10.1158/1078-0432.ccr-19-3919. 10.1158/1078-0432.CCR-19-3919 32019857

[32] Shouval R, Bondi O, Mishan H, Shimoni A, Unger R, Nagler A (2014). Application of machine learning algorithms for clinical predictive modeling: a data-mining approach in SCT. Bone Marrow Transplant.

[33] Shaw BE, Logan BR, Spellman SR, Marsh S, Robinson J, Pidala J (2018). Development of an unrelated donor selection score predictive of survival after HCT: donor age matters most. Biol Blood Marrow Transplant.

[34] Karam E, Laporte J, Solomon SR, Morris LE, Zhang X, Holland HK (2019). Who is a better donor for recipients of allogeneic hematopoietic cell transplantation: a young HLA-mismatched haploidentical relative or an older fully HLA-matched sibling or unrelated donor?. Biol Blood Marrow Transplant.

[35] Shimoni A, Labopin M, Finke J, Ciceri F, Deconinck E, Kröger N, (2019). Donor selection for a second allogeneic stem cell transplantation in AML patients relapsing after a first transplant: a study of the Acute Leukemia Working Party of EBMT. Blood Cancer J..

[36] Bastida JM, Cabrero M, Lopez-Godino O, Lopez-Parra M, Sanchez-Guijo F, Lopez-Corral L (2015). Influence of donor age in allogeneic stem cell transplant outcome in acute myeloid leukemia and myelodisplastic syndrome. Leuk Res.

[37] Seo S, Kanda J, Atsuta Y, Uchida N, Ohashi K, Fukuda T (2015). The impact of donor age on outcome after unrelated bone marrow transplantation: comparison with unrelated cord blood transplantation. Blood.

[38] Rezvani AR, Storer BE, Guthrie KA, Schoch HG, Maloney DG, Sandmaier BM (2015). Impact of donor age on outcome after allogeneic hematopoietic cell transplantation. Biol Blood Marrow Transplant.

[39] Moons KGM, Altman DG, Reitsma JB, Ioannidis JPA, Macaskill P, Steyerberg EW (2015). Transparent reporting of a multivariable prediction model for individual prognosis or diagnosis (TRIPOD): explanation and elaboration. Ann Intern Med.

[40] Collins GS, Reitsma JB, Altman DG, Moons KGM (2015). Transparent Reporting of a multivariable prediction model for Individual Prognosis Or Diagnosis (TRIPOD): The TRIPOD statement. Ann Intern Med.

[41] Lim AB, Roberts AW, Mason K, Bajel A, Szer J, Ritchie DS (2015). Validating the allogeneic stem cell transplantation disease risk index: Sample size, follow-up, and local data are important. Transplantation.

[42] Törlén J, Remberger M, Le Blanc K, Ljungman P, Mattsson J (2017). Impact of pretransplantation indices in hematopoietic stem cell transplantation: knowledge of center-specific outcome data is pivotal before making index-based decisions. Biol Blood Marrow Transplant.

[43] Xhaard A, Porcher R, Chien JW, de Latour RP, Robin M, Ribaud P (2008). Impact of comorbidity indexes on non-relapse mortality. Leukemia.

[44] Shouval R, Fein JA, Shouval A, Danylesko I, Shem-Tov N, Zlotnik M (2019). External validation and comparison of multiple prognostic scores in allogeneic hematopoietic stem cell transplantation. Blood Adv.

[45] Justice AC (1999). Assessing the Generalizability of Prognostic Information. Ann Intern Med.

