# Supplementary material for: A novel Iowa–Mayo validated composite risk assessment tool for allogeneic stem cell transplantation survival outcome prediction
Source: Blood Cancer J. 2021 Nov 20;11(11):183. doi: 10.1038/s41408-021-00573-6 (PMC8606004; doi:10.1038/s41408-021-00573-6)

<u>Supplementary figure 1:</u> Comparison of predicted and observed DFS and OS probabilities among the testing and training cohorts

A: Predicted Vs observed probability of 2-year disease-free survival in the training and testing cohorts, respectively

B: Predicted Vs observed probability of 2-year overall survival in the training and testing cohorts, respectively

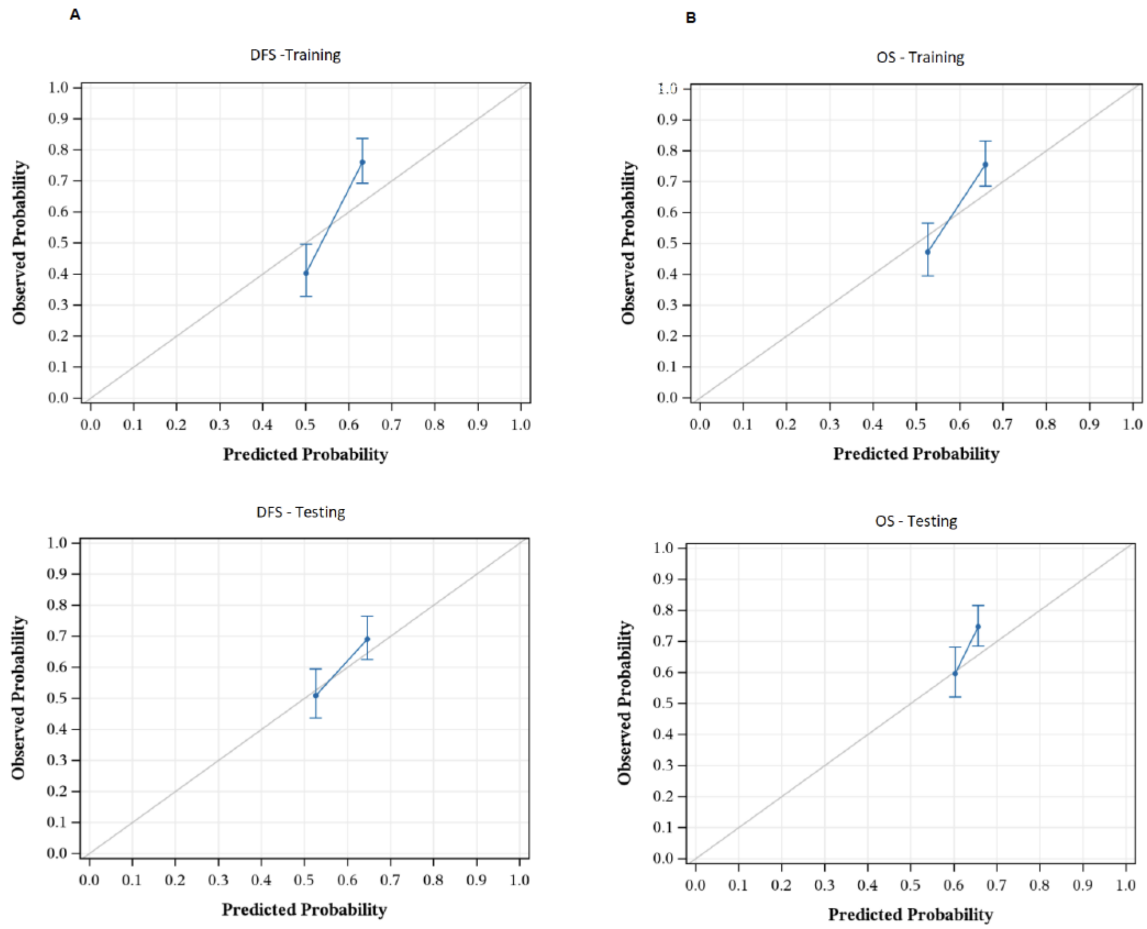

Supplement: Supplementary file 1 — supplementary figure [file 41408_2021_573_MOESM1_ESM.pdf]
